# *Trehalase* Regulates Neuroepithelial Stem Cell Maintenance and Differentiation in the *Drosophila* Optic Lobe

**DOI:** 10.1371/journal.pone.0101433

**Published:** 2014-07-08

**Authors:** Xi Chen, Yaru Quan, Hongbin Wang, Hong Luo

**Affiliations:** 1 School of Life Sciences, Tsinghua University, Beijing, China; 2 Institute for Biological Product Control, National Institutes for Food and Drug Control, Beijing, China; Duke-NUS Graduate Medical School Singapore, Singapore

## Abstract

As one of the major hydrolases in *Drosophila*, trehalase (Treh) catalyzes the hydrolysis of trehalose into glucose providing energy for flight muscle activity. Treh is highly conserved from bacteria to humans, but little is known about its function during animal development. Here, we analyze the function of *Treh* in *Drosophila* optic lobe development. In the optic lobe, neuroepithelial cells (NEs) first divide symmetrically to expand the stem cell pool and then differentiate into neuroblasts, which divide asymmetrically to generate medulla neurons. We find that the knockdown of *Treh* leads to a loss of the lamina and a smaller medulla. Analyses of *Treh* RNAi-expressing clones and loss-of-function mutants indicate that the lamina and medulla phenotypes result from neuroepithelial disintegration and premature differentiation into medulla neuroblasts. Although the principal role of Treh is to generate glucose, the *Treh* loss-of-function phenotype cannot be rescued by exogenous glucose. Thus, our results indicate that in addition to being a hydrolase, Treh plays a role in neuroepithelial stem cell maintenance and differentiation during *Drosophila* optic lobe development.

## Introduction

**Figure 1:**
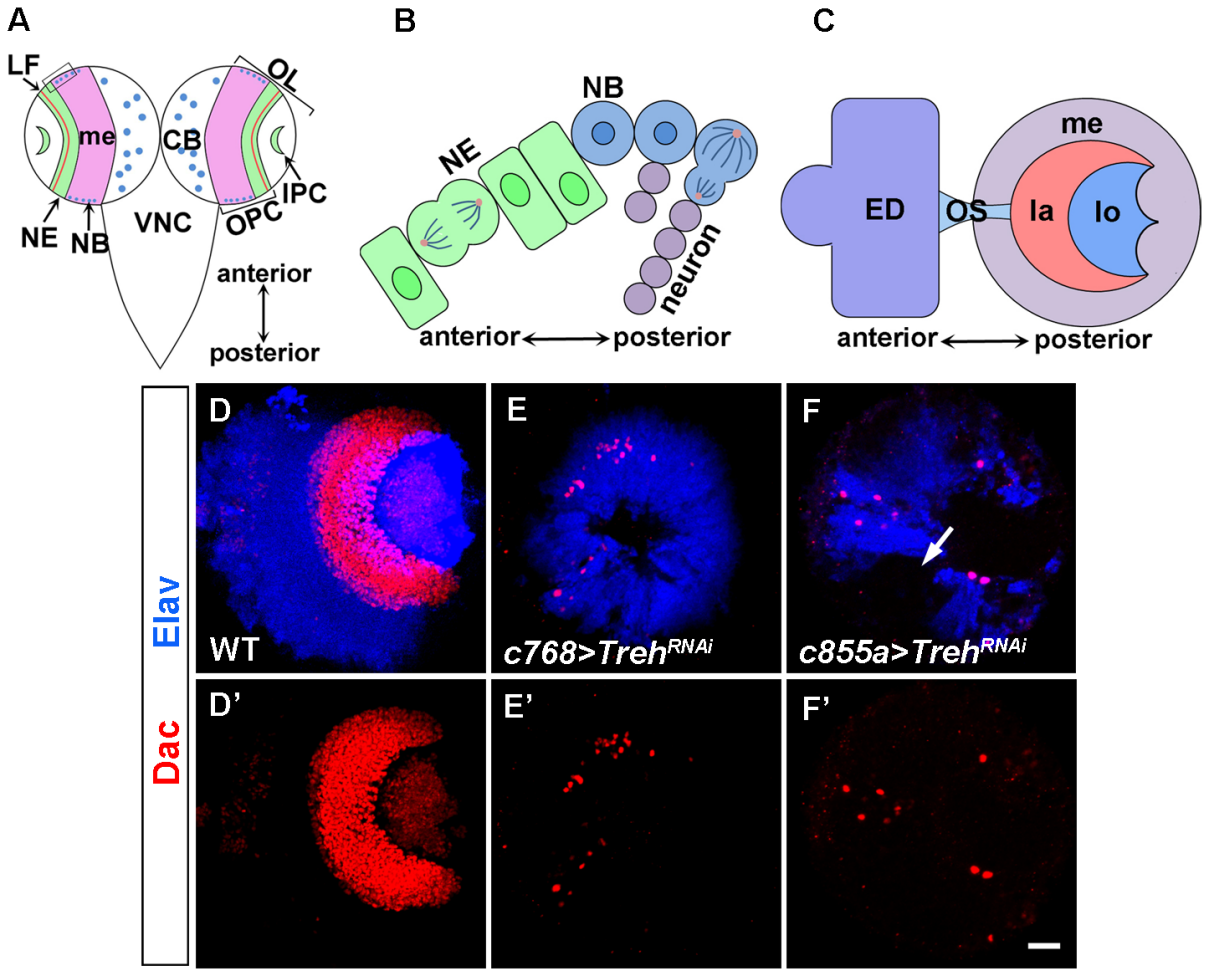
*Treh* is essential for lamina and medulla development. (A) Schematic diagram of the larval CNS. OL: optic lobe; CB: central brain; OPC: outer proliferation center; IPC: inner proliferation center; LF: lamina furrow; me: medulla; NE: neuroepithelial cell; NB: neuroblast in the optic lobe and central brain; VNC: ventral nerve cord. (B) Magnified view of boxed region in (A). NEs in the medial region of the OPC differentiate into medulla NBs; the NBs divide asymmetrically to generate a neuroblast daughter and a smaller ganglion mother cell (GMC) that generates medulla neurons. (C) Lateral view of the optic lobe showing the visual processing neuropils, the medulla (me), lamina (la) and lobula complex (lo). The optic lobe is connected with the eye imaginal disc (ED) through the optic stalk (OS). (D-F) Brains dissected from late-third instar larvae were stained with Dac and Elav to visualize the lamina and medulla, respectively. (D) Wild-type brains have a crescent-shaped lamina and a dome-shaped medulla. (E) *c768-Gal4/UAS-Treh^RNAi^* brains do not have a lamina. (F) *c855a-Gal4/UAS-Treh^RNAi^* brains do not have a lamina, but have an underdeveloped medulla with regions that contained no differentiated neurons (indicated by arrow). Scale bar: 20 µm.

The optic lobe of the *Drosophila* brain is the visual processing center, which contains four neuropils: the lamina, medulla, lobula and lobula plate (Figure 1C) [1]. The optic lobe originates from an embryonic optic placode and in the larval stages develops as two proliferation centers: the outer proliferation center (OPC) and the inner proliferation center (IPC) (Figure 1A) [2], [3]. The IPC generates lobula complex and inner medulla neurons, whereas the OPC gives rise to lamina and outer medulla neurons [1].

During early larval development, neuroepithelial cells (NEs) of the optic lobe proliferate by symmetric division, thereby expanding the stem cell pool. At late second instar, the NEs on the medial edge of the OPC begin to differentiate into medulla neuroblasts (NBs). These neuroblasts undergo asymmetric division producing a neuroblast daughter and a smaller ganglion mother cell (GMC) that divides once to generate two medulla neurons (Figure 1B) [4]–[7]. This proliferation and differentiation pattern closely resembles that of neural progenitor cells in the developing vertebrate brain [8]–[10]. In the past few years, a number of researchers have used the *Drosophila* optic lobe as a model to analyze the key signaling mechanisms controlling neural stem cell maintenance and the transition from symmetric to asymmetric division. Several signals have been identified that regulate the maintenance and differentiation of neuroepithelial stem cells, including the JAK/STAT, Notch, Fat/Hippo and EGFR pathways [11]–[19].

The *Trehalase* (*Treh*) gene of *Drosophila melanogaster* encodes a highly conserved hydrolase (Figure S1). Treh not only generates energy by hydrolyzing trehalose into two glucose moieties [20], [21], but also acts as a stress-response protein, protecting cell membranes and proteins from damages resulting from high temperatures, freezing and desiccation [22], [23]. However, the role of Treh in animal development has not been well studied.

In this study, we have examined the function of Treh in the development of the *Drosophila* optic lobe. We find that the loss of *Treh* causes neuroepithelial disintegration and premature generation of neuroblasts, leading to severe brain defects, while exogenous glucose cannot rescue the phenotypes. We conclude that Treh controls neuroepithelial stem cell maintenance and suppresses their differentiation into neuroblasts in the *Drosophila* optic lobe.

## Materials and Methods

### Fly stocks

Flies were reared on standard cornmeal food at 25°C unless otherwise indicated. *w^1118^* was used as a wild-type strain. The following transgenic fly lines were used. *UAS-Treh^RNAi^* (Vienna *Drosophila* RNAi Center stock 30730) encodes a *Treh* RNAi construct. *Treh^EY06982^* (Bloomington *Drosophila* Stock Center stock 16775) carries the transposable element P{EPgy2} inserted into the second intron of *Treh*. The *Sb*, Δ*2-3/TM6B* line contains a transposase-encoding gene inserted at 99B on the third chromosome. Gal4 lines used include *c855a-Gal4* [24], *c768-Gal4* [25] and *NP3605-Gal4* [15].

### Genetic crosses

The UAS/GAL4 system was used for overexpression and RNAi experiments [26]. For *Treh* RNAi knockdown, *UAS-Treh^RNAi^* females were crossed with *c768-Gal4*, *c855a-Gal4* or *NP3605-Gal4* males, and the progeny were cultured at 25°C. For *Treh* overexpression, *UAS-Treh* females were crossed to *c768-Gal4* males and then cultured at 31°C, at which temperature the Gal4 has a higher activity.

To induce clones that express *Treh* RNAi, *UAS-Treh^RNAi^* females were crossed with *y w hsFlp1/Y; actin<y+<Gal4, UAS-nGFP* males, the larval progeny were subjected to a one-hour heat shock at 38°C at approximately 48 hours after larval hatching (ALH), then cultured at 25°C until late-third instar before dissection.

### Immunohistochemistry

Larval brain staining was performed as previously described [18]. The following primary antibodies were used: guinea pig anti-Deadpan (1∶1000, Luo lab), rat anti-Miranda (1∶1000, a gift from Chris Doe), guinea pig anti-Numb (1∶1000, a gift from James Skeath), rabbit anti-activated caspase-3 (9661S, 1∶200, Cell Signaling Technology), rabbit anti-pAkt (D9E, 1∶100, Cell Signaling Technology), rabbit anti-phospho-Histone H3 (06-570, 1∶500, Upstate Biotechnology), mouse anti-Discs large [4F3, 1∶100, Developmental Studies Hybridoma Bank (DSHB)], mouse anti-Dachshund (mAbdac2-3, 1∶100, DSHB), rat anti-Elav (7E8A10, 1∶100, DSHB), mouse anti-β-Tubulin (E7, 1∶20, DSHB), rabbit anti-DE-Cadherin (sc-33743, 1∶100, Santa Cruz Biotechnology), rabbit anti-aPKC (sc-216, 1∶1000, Santa Cruz). The secondary antibodies used were: Alexa Fluo-488 goat anti-rabbit (1∶200, Molecular Probes); Cy3-conjugated donkey anti-mouse (1∶200), Cy3-conjugated goat anti-rabbit (1∶200), Cy5-conjugated donkey anti-rat (1∶200), Cy5-conjugated goat anti-rabbit (1∶200) and Cy5-conjugated donkey anti-guinea pig (1∶200) (Jackson ImmunoResearch Lab).

Confocal images were obtained by Olympus FV500 (60 x objective, N.A.1.4) and Nikon A1R MP (60 x (WI) objective, N.A.1.27) confocal microscopes, and processed with Imaris (Bitplane) and Adobe Photoshop CS software.

### RNA preparation and quantitative real-time PCR

Total RNA was isolated from dissected larval central nervous system (CNS) using the TRIzol reagent (Invitrogen) as previously described [27], and complementary DNA (cDNA) was reverse-transcribed using SuperScript™ III Reverse Transcriptase (Invitrogen) according to the manufacturer's instruction. Quantitative real-time PCR (qRT-PCR) was performed by a BioRad iQ5 instrument using a RealMasterMix kit (SYBR Green, Tiangen Biotech), and mRNA levels were normalized against the housekeeping gene *Ribosomal protein 49 (Rp49)*. The primer sequences were listed in Table S1.

### Generation of *Treh* mutant alleles by P-element imprecise excision

*Treh^EY06982^* flies are viable and display no visible defects. We set to create stronger loss-of-function *Treh* alleles by imprecise excision of the P-element. To mobilize the P-element, *Treh^EY06982^* females were crossed with *Sb*, Δ*2-3/TM6B* males; the F1 progeny were crossed with *CyO/Sp* flies. The white-eyed F2 progeny were individually crossed to *CyO/Sp* flies to establish mutant lines.

The lesions in *Treh* mutant alleles were determined by PCR analyses of genomic DNAs isolated from homozygous *Treh* mutant larvae.

### Transgenic flies

To generate *UAS-Treh* flies for overexpression, *Treh* cDNA (isoform-E) was amplified by PCR using primers listed in Table S1 and cloned into the pUAST vector. The pUAST-*Treh* plasmid, together with a helper plasmid that expresses a transposase, was co-injected into *w^1118^* stage-2 embryos according to the standard germline transformation procedure [28].

## Results

### *Trehalase* is essential for lamina and medulla development

Our previous study showed that *Treh* mRNA is expressed in the optic lobe NEs [27]. To study the function of *Treh*, we knocked down *Treh* activity by RNA interference (RNAi) using either *c768-Gal4* or *c855a-Gal4*, two drivers that are active in the optic lobe NEs from the first instar stage onward [4], [18]. Late-third instar larval brains were stained for markers that visualize the structure of the brain. In the wild type, the crescent-shaped lamina can be visualized by staining for Dachshund (Dac), while Elav staining labels neurons and reveals the dome-shaped medulla (Figure 1D, D'). In *Treh* RNAi brains, the lamina was absent, with only a small number of cells present (Figure 1E, E', n = 15; F, F', n = 13); in addition, the medulla was underdeveloped which contained regions with no differentiated neurons (Figure 1F, indicated by arrow).

Because *Treh* RNAi driven by *c768-Gal4* or *c855a-Gal4* led to comparatively small eye imaginal discs, which could affect lamina development [29], [30], we targeted *Treh* RNAi more specifically in the optic lobe. Under the control of *NP3605-Gal4* [15], *Treh* RNA was knocked down in the OPC but not in the eye disc cells; this RNAi knockdown also led to the loss of the lamina and a small medulla (data not shown). Thus, we conclude that *Treh* is essential for lamina and medulla development.

### *Treh* is required for neuroepithelial maintenance in the optic lobe

Since both lamina and medulla neurons originate from optic lobe NEs, the above results suggest that *Treh* may function during neuroepithelial expansion. Thus, we examined NE proliferation and differentiation in *Treh* mutant brains at different larval stages. NEs are columnar cells which are arranged regularly in the lateral region of the OPC neuroepithelium. They express epithelial cell markers PatJ, atypical PKC (aPKC) and Crumbs on the apical domain, and adherens junction proteins DE-Cadherin (DE-Cad) and Armadillo.

**Figure 2:**
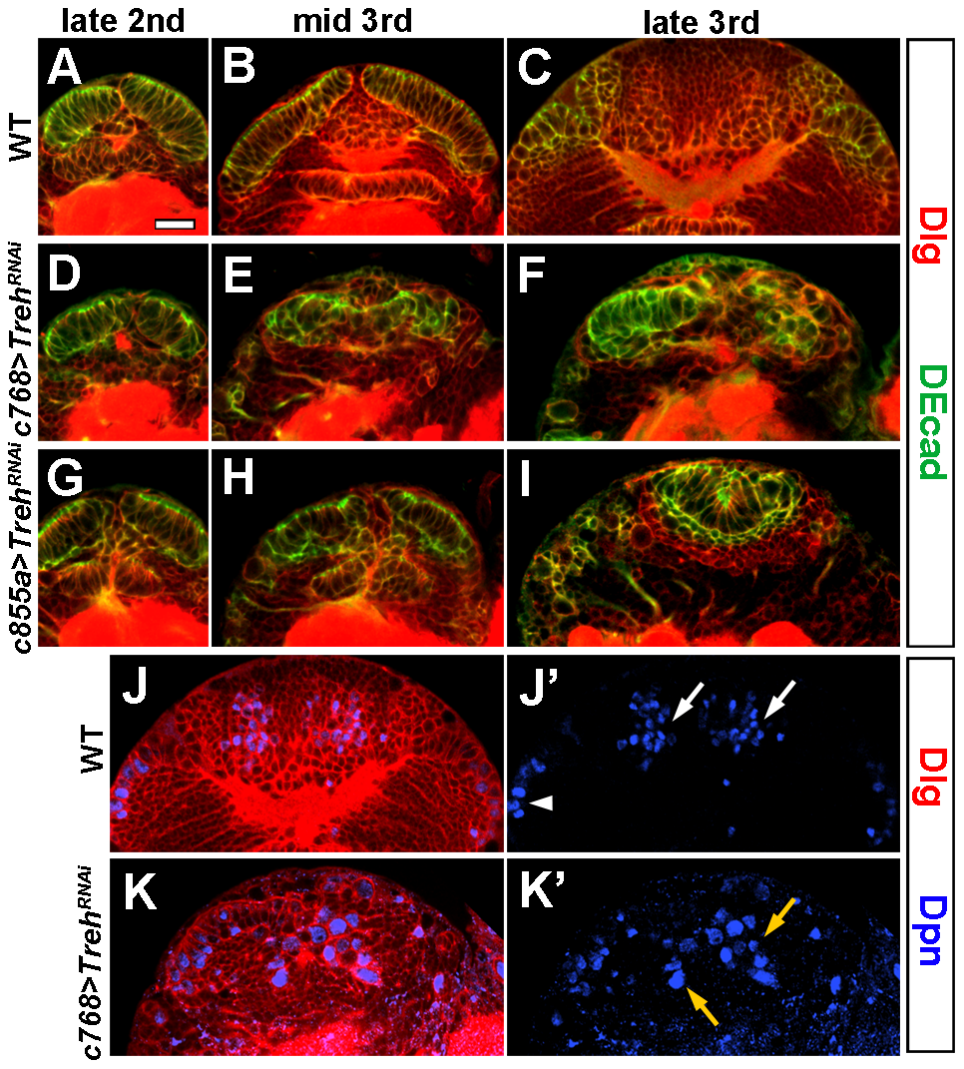
*Treh* regulates neuroepithelial cell maintenance and differentiation in the optic lobe. (A-I) Time courses of neuroepithelial growth and expansion. (A-C) Wild-type brains at late-second (A), mid-third (B) and late-third instar (C). (D-F) *c768-Gal4/UAS-Treh^RNAi^* brains at late-second (D), mid-third (E) and late-third instar (F). The OPC neuroepithelium was normal at late-second instar (D), but became gradually disintegrated from mid-third (E) to late-third instar stages (F). (G-I) *c855a-Gal4/UAS-Treh^RNAi^* brains at late-second (G), mid-third (H) and late-third instar (I). The OPC neuroepithelium began to disintegrate around mid-third instar. (J, K) *Treh* RNAi brains had some enlarged, rounded cells that were Dpn^+^ and localized in the medulla cortex (K, K' indicated by yellow arrows), whereas wild-type brains have medulla neuroblasts localized on the medial surface of the optic lobe (J, J', indicated by white arrowhead). White arrow indicates IPC neuroblasts, which were not analyzed in this study. Scale bar: 20 µm.

In the wild type, NEs divide symmetrically during the first- and second-instar larval stages and then start to differentiate into neuroblasts, such that the NEs reach a maximal number by the mid-third instar stage. *Treh* RNAi knockdown using *c768-Gal4* did not cause obvious defects in late-second-instar larval brains, as they had a similar number of morphologically normal NEs to wild-type brains (Figure 2D, n = 18, compare with A). However, around the mid-third instar stage, *Treh* mutant NEs became disintegrated, and changed cell morphology (Figure 2E, n = 21); and by late-third instar, few NEs were left in the optic lobe, while some enlarged, rounded cells appeared in the medulla cortex (Figure 2F, n = 47). These enlarged cells expressed the neuroblast markers Dpn (Figure 2K, K', n = 27, indicated by yellow arrow) and Mira (not shown), suggesting that they were ectopic neuroblasts that might have originated from the disintegrated neuroepithelium. *Treh* RNAi knockdown using *c855a-Gal4* similarly caused neuroepithelial disintegration starting from the mid-third instar stage (Figure 2G, n = 12; H, n = 22; I, n = 50).

The above results demonstrate that *Treh* is required for neuroepithelial maintenance, and the loss of *Treh* function leads to the loss of NEs.

### *Treh* suppresses the differentiation of neuroepithelial cells

To further characterize Treh mutant NEs in the optic lobe, we conducted cell lineage analyses by inducing flip-out clones that expressed Treh RNAi.

**Figure 3:**
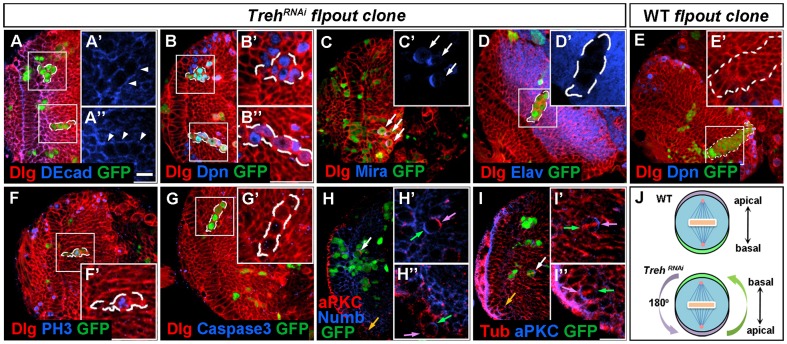
*Treh* suppresses the differentiation of neuroepithelial cells. Late-third instar larval brains were stained with the antibodies indicated and flip-out clones expressing *Treh* RNAi were marked by GFP and dashed lines. (A) Cells in *Treh* RNAi clones in the medulla cortex were large and rounded (indicated by white arrowhead). (B) Multiple cells in each *Treh* RNAi clone expressed Dpn. (C) *Treh* RNAi mutant cells had asymmetric Mira localization in the cell cortex. (D) *Treh* RNAi clones generated only a limited number of neurons as revealed by Elav staining. (E) A wild-type control clone had a large lineage with some neuroblasts localized on the medial surface of the OPC. (F) *Treh* RNAi mutant cells underwent proliferation as revealed by PH3 staining. (G) No apoptotic cell death of *Treh* RNAi mutant cells was detected by activated caspase-3 staining. (H) Ectopic neuroblasts in *Treh* RNAi clones had asymmetric aPKC and Numb localization at opposite poles. The apical and basal poles (H') were reversed as compared with wild-type medulla neuroblasts (H”). (I) Tubulin staining of *Treh* RNAi mutant cells revealed that the spindle was aligned along the apicobasal axis. In (H) and (I), white and yellow arrows indicate *Treh* RNAi mutant neuroblast and normal medulla neuroblast, respectively; purple and green arrows indicates apical and basal pole, respectively. (J) Schematic showing *Treh* RNAi mutant neuroblasts with a reversal of apical and basal poles as compared with normal medulla neuroblasts. Scale bar: 20 µm.

Two classes of clones were observed. The *Treh* RNAi clones that remained in the OPC neuroepithelium did not change epithelial cell identity as revealed by DE-Cad and aPKC staining (data not shown). However, the clones were more frequently found in the medulla cortex (79.5%, n = 39), suggesting that *Treh* mutant cells were unstable within the neuroepithelium and extruded basally into the medulla. The extruded cells changed their morphology to large, rounded cells, which were easily distinguished from their wild-type neighbors (Figure 3A, n = 27, indicated by white arrowhead). These mutant cells had an average diameter of 9.5 µm, which is comparable to the size of wild-type medulla neuroblasts. The *Treh* RNAi clones had a limited number of cells (7 cells on average), however, about two thirds of the cells expressed Dpn (Figure 3B, n = 41) and had asymmetric Mira localization in the cell cortex (Figure 3C, n = 16); in contrast, wild-type clones had a large number of cells with a few neuroblasts localized on the medial surface of the optic lobe (Figure 3E, n = 24). Analyses of mitotic cells by anti-phospho-Histone 3 (PH3) staining showed that the *Treh* mutant cells were able to undergo proliferation (Figure 3F, n = 13, 70%); and they were alive as revealed by the lack of activated caspase-3 staining (Figure 3G, n = 6).

Neuroblasts divide asymmetrically, with proteins localized at the apical or the basal cell cortex, for example, aPKC, an apical component of the Par protein complex, and Numb, a basal protein, show asymmetric cortical crescents during metaphase. We examined the division patterns of *Treh* mutant neuroblasts by checking the expression of aPKC and Numb. In *Treh* RNAi clones, the ectopic neuroblasts displayed asymmetric localizations of aPKC and Numb at the opposite poles (Figure 3H, n = 7). However, the apical and basal poles were reversed as compared with wild-type medulla neuroblasts (Figure 3H-J). Typically, medulla NBs have the apical and basal poles facing the surface and the interior of the brain, respectively (Figure 3H'', 3I'', indicated by yellow arrow); in contrast, 84.2% (n = 38) of the *Treh* mutant neuroblasts had the apical pole facing the interior and the basal pole facing the brain surface (Figure 3H', 3I'). We noticed that at metaphase, the spindle was still aligned along the apicobasal axis, which would allow the asymmetric division to occur (Figure 3I, n = 14).

However, *Treh* mutant NBs in the anaphase or telophase were rarely observed, and the clones generated few neurons as shown by Elav staining (Figure 3D, n = 19, compare with the large lineage of a wild-type clone in 3E). These data demonstrate that when *Treh* is knocked down in the NEs, the cells extrude into the medulla, and prematurely differentiate into neuroblasts, although the generation of neurons is limited.

### Effects of *Treh* loss-of-function mutations on optic lobe development

**Figure 4:**
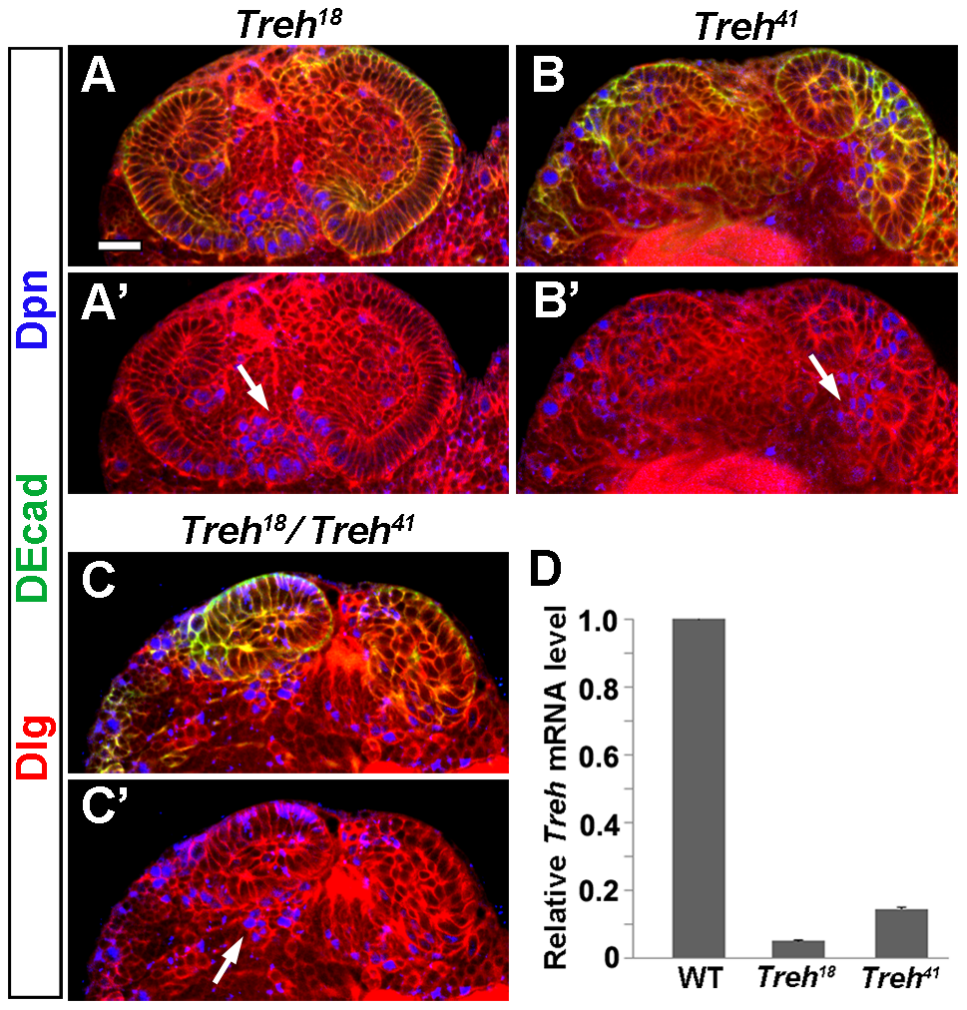
*Treh* loss-of-function mutations cause neuroepithelial disintegration and premature neuroblast formation. (A, B) *Treh^18^* and *Treh^41^* homozygous late-third-instar larval brains had partly disintegrated OPC neuroepithelia, with some NEs transformed to rounded cells that expressed Dpn (indicated by arrow). (C) *Treh^18^/Treh^41^* late-third-instar larval brains also had disintegrated NEs and premature formation of NBs (indicated by arrow). (D) Quantification of *Treh* mRNA levels in wild type and *Treh* mutants by real-time PCR analysis. Scale bar: 20 µm.

We generated two *Treh* loss-of-function mutations using P-element imprecise excision and mapped the lesions by PCR at the molecular level (Figure S2C). In *Treh^18^*, about 860 bp of the second intron of *Treh* was deleted, whereas in *Treh^41^*, there was no deficiency in the *Treh* gene itself, but at least 38 bp of the P-element was left in the intron of *Treh* after imprecise excision, which could influence the transcription of *Treh* (Figure S2B). Indeed, *Treh* transcript levels in *Treh^18^* and *Treh^41^* homozygous animals were dramatically decreased to 5% and 14% that in wild type, respectively (Figure 4D). These results indicate *Treh^18^* and *Treh^41^* are indeed loss-of-function alleles.

Both *Treh^18^* and *Treh^41^* homozygous animals die at the late-third instar or pupal stages; however, 2% and 6% of them, respectively, survived to adulthood. In brains dissected from *Treh^18^* homozygous late-third instar larvae, the OPC neuroepithelium became partly disintegrated, and the NEs changed cell morphology to rounded cells, which expressed Dpn (Figure 4A, n = 8). *Treh^41^* homozygotes showed consistent but even more severe phenotypes, as the neuroepithelium was largely disintegrated (Figure 4B, n = 24). To eliminate the possibility that the imprecise excision led to deficiency of unrelated genes, we did a complementation test by combining these two mutant alleles together. As expected, *Treh^18^* failed to complement *Treh^41^*, and *Treh^18^/Treh^41^* animals also showed disorganized NEs and premature formation of NBs (Figure 4C, n = 17). These *Treh* mutant results confirm that *Treh* is required for NE maintenance and suppression of NE differentiation into NBs.

### *Treh* is not sufficient for neuroepithelial development in the optic lobe

**Figure 5:**
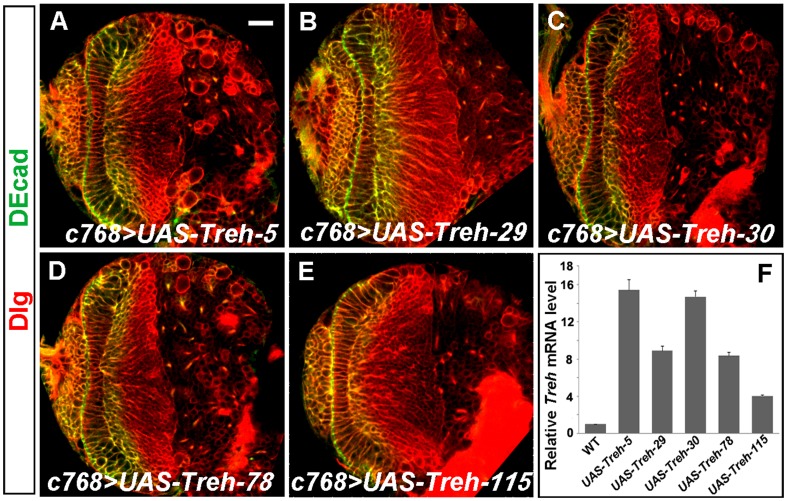
*Treh* overexpression does not affect optic lobe development. (A-E) Late-third instar larval brains expressing five different *UAS-Treh* lines under the control of *c768-Gal4*. Overexpression of *Treh* did not cause defects in the brain; and the proliferation and differentiation of NEs was normal. (F) Quantification of *Treh* mRNA levels in wild-type and *c768-Gal4/UAS-Treh* larval CNS by real-time PCR analysis. Scale bar: 20 µm.

Since loss of *Treh* function caused neuroepithelial disintegration and premature formation of NBs, we tested whether *Treh* overexpression may cause NE overproliferation or delay the NE-to-NB transition. We generated a number of *UAS-Treh* lines carrying *Treh* cDNA. Different *UAS-Treh* lines were expressed using *c768-Gal4* and late-third instar larval brains were examined. Quantitative PCR analyses indicated that *Treh* mRNA levels increased 4-16 times that in wild-type brains (Figure 5F); however, none of these *Treh*-overexpressing brains had any defects in NE proliferation or differentiation (Figure 5A-E), and the brains were quite normal. Thus, the overabundance of *Treh* does not influence optic lobe development.

### *Treh* regulation of neuroepithelial maintenance is independent of hydrolase function

Since Treh hydrolyzes trehalose into glucose, we wondered if the disintegration of the OPC neuroepithelium and premature generation of NBs in *Treh* mutant brains were due to a lack of glucose. To test this hypothesis, we carried out glucose feeding experiments by rearing the mutant animals on standard cornmeal food supplemented with 10% or 20% glucose. Neither glucose culture condition could rescue the high mortality rate of *Treh^18^* and *Treh^41^* homozygous mutants. In addition, the brain defects in animals expressing *Treh* RNAi under the control of *c768-Gal4* were not rescued by the addition of 10% glucose; and clones expressing *Treh* RNAi exhibited similar defects on normal and glucose-added food. These results indicate that exogenous glucose cannot compensate for the lack of Treh and suggest that Treh may regulate neuroepithelial maintenance and differentiation independent of its hydrolase activity.

## Discussion

In this study, we have shown that trehalase plays an important role in the maintenance of neuroepithelial stem cells in the *Drosophila* larval optic lobe. Loss of *Treh* function causes neuroepithelial disintegration and premature neuroblast formation. The deficiency in neuroepithelial growth results in an insufficient number of precursor cells for the lamina and medulla neuropils, leading to severe brain defects. In addition, the prematurely formed mutant neuroblasts divide with a reversed apicobasal polarity, which may also affect the neuroblast's ability to generate medulla neurons.

The *Treh* loss-of-function phenotype is reminiscent of JAK/STAT mutants [11], [17], which also exhibit early depletion of the OPC neuroepithelium and premature neuroblast formation. This phenotypic similarity suggests that Treh may be a downstream effector of the JAK/STAT pathway. Indeed, *Treh* expression is positively regulated by JAK/STAT [27], possibly through direct STAT92E binding to a putative enhancer containing three STAT92E binding sites. Although our preliminary ChIP assay showed that there was no obvious enrichment of STAT92E binding to *Treh* sequences as compared with the control IgG, this model of transcriptional activation remains to be verified. *Treh* overexpression did not cause any phenotype in the optic lobe; one explanation is that *Treh* is among a number of genes that respond to JAK/STAT signaling in the optic lobe.

The well-known function of Treh is the hydrolysis of trehalose, which is the principal hemolymph sugar in *Drosophila* [31]–[33]. Loss of *Treh* function could result in a lack of intracellular glucose in the optic lobe NEs, which might affect neuroepithelial stem cell maintenance. However, we found that the *Treh* loss-of-function phenotypes cannot be suppressed by exogenous glucose. Alternatively, loss of *Treh* may cause an accumulation of trehalose in the NEs, which might alter the nutritional state in the NEs. The insulin receptor (InR)/PI3 Kinase pathway is nutrition-dependent, and has been shown to be required for thoracic ventral nerve cord (tVNC) neuroblast activation and proliferation [34]. We tested whether loss of *Treh* might affect the insulin signaling pathway in the optic lobe NEs, but found that this pathway is not active in wild-type or *Treh* mutant NEs as accessed by phosphorylated Akt (pAkt) expression (data not shown). From these analyses, we infer that Treh may target other pathways or processes to regulate neuroepithelial maintenance and differentiation, rather than functions solely as a hydrolase in the NEs.

In mammals, trehalose is not the principal sugar in the blood and Treh functions as a hydrolase mainly during the active transport of glucose to the kidney and intestine [35]. However, mammalian trahalases may also have hydrolase-independent functions; and perhaps they play a role in the maintenance and differentiation of neural stem cells during mammalian brain development.

## Supporting Information

Figure S1 **A multiple sequence alignment of Treh proteins from different species.** Treh is highly conserved among *Homo sapiens*, *Macaca mulatta*, *Rattus norvegicus*, *Mus musculus* and *Drosophila melanogaster*. The conserved and similar amino acid residues are shaded in black and grey according to the degree of similarity.
(TIF)

Figure S2 **Determination of lesions in** ***Treh^18^*** **and** ***Treh^41^*** **by PCR.** (A) Partial genomic sequence of *Treh* and the insertion site of the P{EPgy2} element. (B) Schematic diagrams showing the lesions in *Treh^18^* and *Treh^41^*. In *Treh^18^*, about 860 bp of the second intron of *Treh* (indicated by grey line) were deleted upstream of the insertion site, whereas *Treh^41^* contains at least 38 bp of the P-element (indicated by red line) in the second intron of *Treh*. (C) Determining the lesions in *Treh^18^* and *Treh^41^*. Genomic DNA from homozygous mutant larvae was amplified using primer pairs shown, and the primer sequences were list in Table S1. The long line in black and red indicates *Treh* genomic DNA and P{EPgy2} DNA, respectively. Arrows above the lines indicate forward primers, while the ones below means reverse primers; black arrows indicate *Treh* primers and red ones indicate P{EPgy2} primers.
(TIF)

Table S1 **List of Primer Sequences.**
(DOCX)
