# Supplementary figures and images for: Trehalase Regulates Neuroepithelial Stem Cell Maintenance and Differentiation in the Drosophila Optic Lobe
Source: PLoS One. 2014 Jul 8;9(7):e101433. doi: 10.1371/journal.pone.0101433 (PMC4086926; doi:10.1371/journal.pone.0101433)

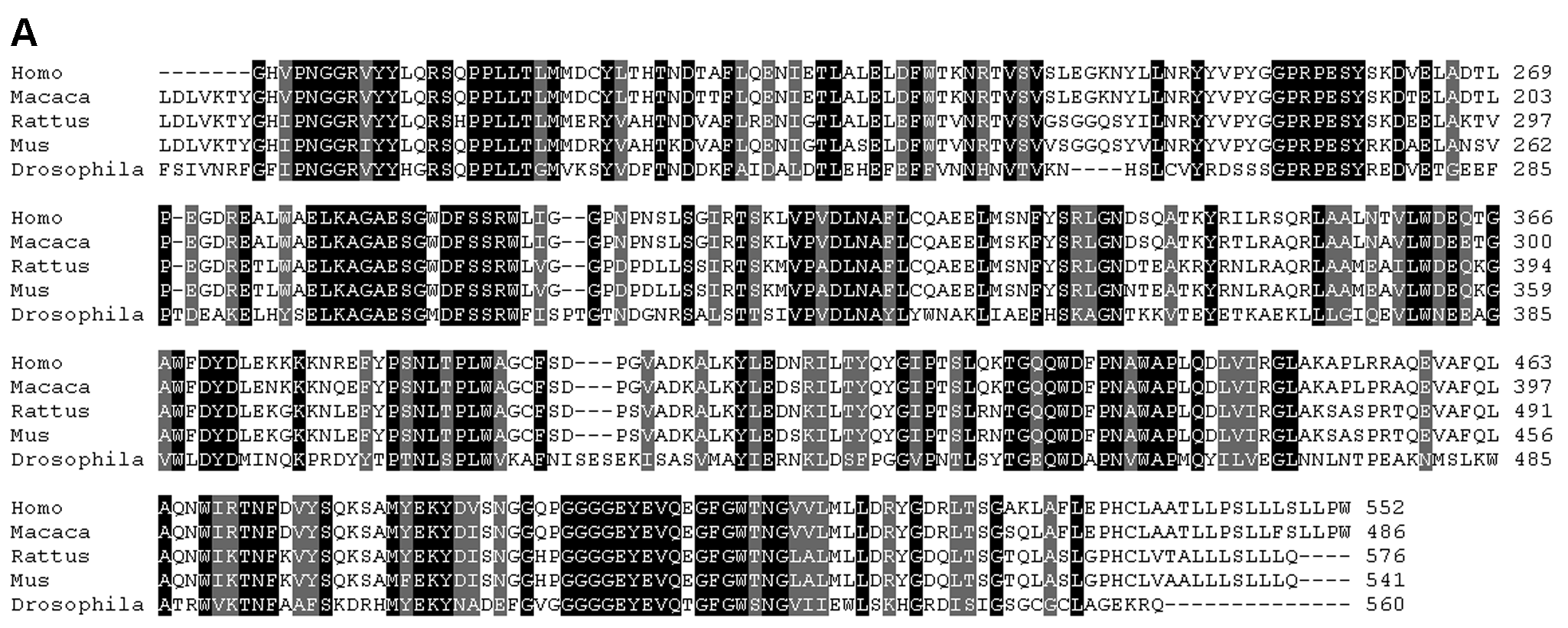

Supplement: Figure S1 — A multiple sequence alignment of Treh proteins from different species. Treh is highly conserved among Homo sapiens, Macaca mulatta, Rattus norvegicus, Mus musculus and Drosophila melanogaster. The conserved and similar amino acid residues are shaded in black and grey according to the degree of similarity. (TIF) [file pone.0101433.s001.tif]

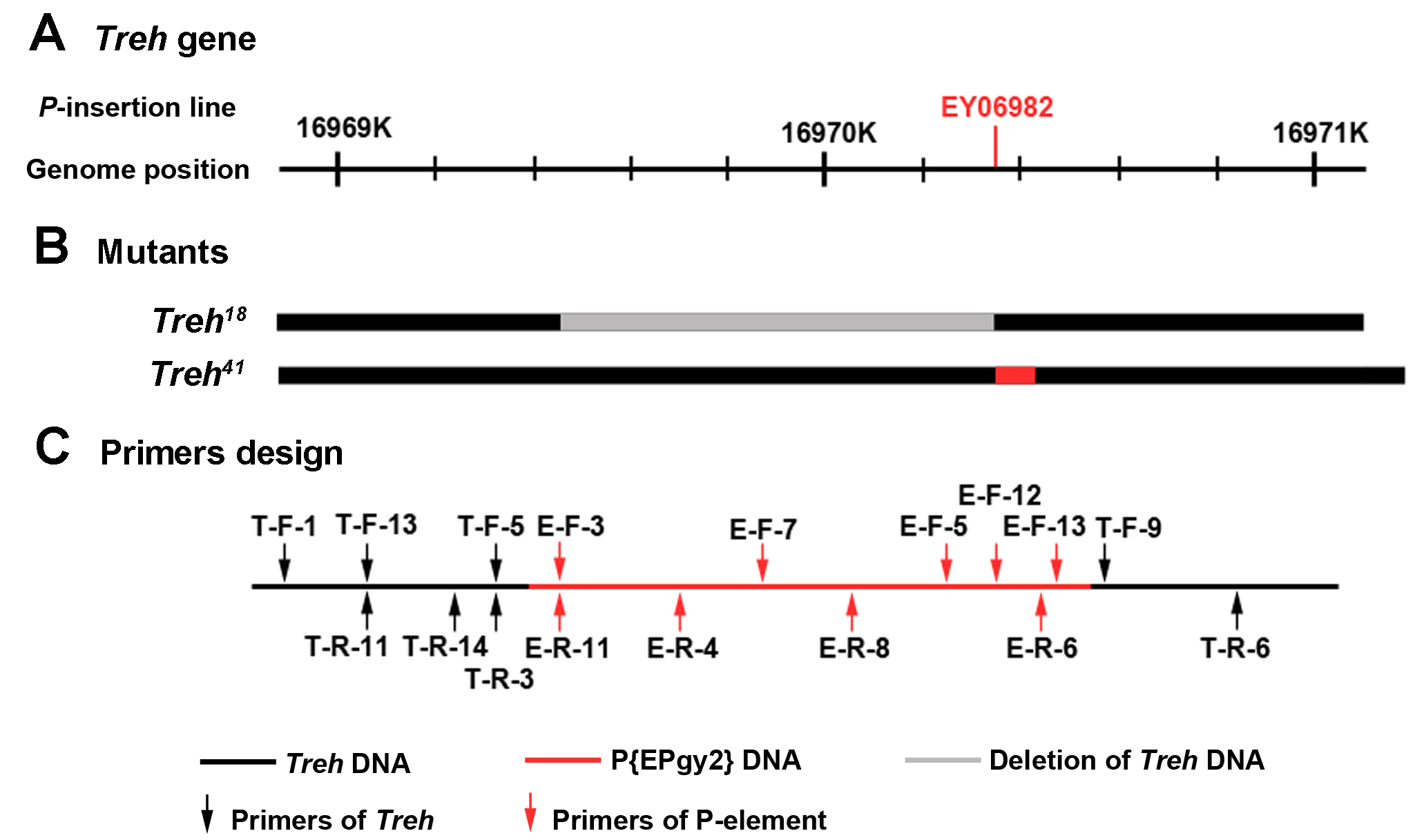

Supplement: Figure S2 — Determination of lesions in Treh18 and Treh41 by PCR. (A) Partial genomic sequence of Treh and the insertion site of the P{EPgy2} element. (B) Schematic diagrams showing the lesions in Treh18 and Treh41. In Treh18, about 860 bp of the second intron of Treh (indicated by grey line) were deleted upstream of the insertion site, whereas Treh41 contains at least 38 bp of the P-element (indicated by red line) in the second intron of Treh. (C) Determining the lesions in Treh18 and Treh41. Genomic DNA from homozygous mutant larvae was amplified using primer pairs shown, and the primer sequences were list in Table S1. The long line in black and red indicates Treh genomic DNA and P{EPgy2} DNA, respectively. Arrows above the lines indicate forward primers, while the ones below means reverse primers; black arrows indicate Treh primers and red ones indicate P{EPgy2} primers. (TIF) [file pone.0101433.s002.tif]
