# Supplementary material for: Trehalase Regulates Neuroepithelial Stem Cell Maintenance and Differentiation in the Drosophila Optic Lobe
Source: PLoS One. 2014 Jul 8;9(7):e101433. doi: 10.1371/journal.pone.0101433 (PMC4086926; doi:10.1371/journal.pone.0101433)
Supplement: Table S1 — List of Primer Sequences. (DOCX) [file pone.0101433.s003.docx]

| Category | Primer name | Sequence |
| --- | --- | --- |
| Overexpression stocks | Treh OE F-#1 | CGCGGATCCGAATTCGCGACATCAGGAAACAACAG |
|  | Treh OE R-#2 | CCCACATTGTGGTACCGTTCTGGCCTGGCTACAGAT |
| qRT-PCR analysis | Treh OE RT F-#3 | CTACCGAGAGGATGTGGAGAC |
|  | Treh OE RT R-#4 | AATGGAGGTGGTGCTCAGAG |
|  | Treh M RT F-#1 | CGCTCATCAACTGCTCATCC |
|  | Treh M RT R-#2 | GCTGTAACTGTGGTGCTGTG |
| Molecular mapping of mutants | Treh M F-#1 | CTTAAAACTGAAGAGCCTTGAATGCCGCTG |
|  | Treh M R-#11 | GCTTTGATTAGCCGTTCAAATGCCGATCG |
|  | Treh M F-#13 | CGATCGGCATTTGAACGGCTAATCAAAGC |
|  | Treh M R-#14 | ATGCAGCCCGATCATGAAGCAACTATTGT |
|  | Treh M F-#5 | ATGTTTTCCAATGCCTGACACGTAGGGCCA |
|  | Treh M R-#3 | TGGCCCTACGTGTCAGGCATTGGAAAACAT |
|  | Treh M F-#9 | CCAACTTGCAAATGCCGAATATGACTTCGC |
|  | Treh M R-#6 | GGTTCACCTGCTTCAAGTCTCGTGAACACA |
|  | EPgy2 F-#3 | AATAACATAAGGTGGTCCCGTCGATAGCCG |
|  | EPgy2 R-#4 | TTTCCCCTTCGAACATCCCCACAAGTAGAC |
|  | EPgy2 F-#5 | TTCTCGAGGTCATCAAGCTTAGGCCTCCAA |
|  | EPgy2 R-#6 | CGAGTGAAAGGAATAGTATTCTGAGTGTCG |
|  | EPgy2 F-#7 | CTGAAGGCGGACATTGACGCTACGTAACGC |
|  | EPgy2 R-#8 | GATCGCCTGGTCGCTTGAGATTCGACTGTA |
|  | EPgy2 F-#12 | ACACAACCTTTCCTCTCAACAA |
|  | EPgy2 R-#11 | CGGCTATCGACGGGACCACCTTATGTTATT |
|  | EPgy2 F-#13 | CTTGCCGACGGGACCACCTTATGTTATT |

**Table S1 List of primer sequences**
